# Correspondence

**Published:** 1913-01

**Authors:** H. W. Cowper


### CORRESPONDENCE.

December 26, 1912.

The Editor BUFFALO MEDICAL JOURNAL:

Dear Sir:—That there are men in general practice who do not realize the necessity of early refraction in cases of strabismus seems to be evident from statements occasionally heard, especially in dispensary work. "Our doctor said not to mind the baby's cross eyes. He said he would outgrow it."

This is very wrong. When an eye witness to turn, no matter how young the child, the refractive condition should be ascertained. It is possible to do this with absolute satisfaction regardless o fthe age. A colleague prescribed lenses for an infant of seven months the other day. The proportion of eyes straightened by lenses before the age of five is very much greater than when the correction is delayed beyond that age. But the important point is that vision is retained. An eye which is constantly turned for a long time loses a great part of its power of vision, and when once lost in this way seldom is much of it regained.

All this will seem elementary to most of your readers, but, there are some who do not have the proper conception of the requirements in these cases, is certain.

Very truly,

H. W. COWPER.

